# *FACT *– a framework for the functional interpretation of high-throughput experiments

**DOI:** 10.1186/1471-2105-6-161

**Published:** 2005-06-28

**Authors:** Felix Kokocinski, Nicolas Delhomme, Gunnar Wrobel, Lars Hummerich, Grischa Toedt, Peter Lichter

**Affiliations:** 1 Molecular Genetics, Deutsches Krebsforschungszentrum, 69115 Heidelberg, Germany; 2 Wellcome Trust Sanger Institute, Wellcome Trust Genome Campus, Hinxton, Cambridge CB10 1HH, UK

## Abstract

**Background:**

Interpreting the results of high-throughput experiments, such as those obtained from DNA-microarrays, is an often time-consuming task due to the high number of data-points that need to be analyzed in parallel. It is usually a matter of extensive testing and unknown beforehand, which of the possible approaches for the functional analysis will be the most informative

**Results:**

To address this problem, we have developed the *Flexible Annotation and Correlation Tool *(FACT). FACT allows for detection of important patterns in large data sets by simplifying the integration of heterogeneous data sources and the subsequent application of different algorithms for statistical evaluation or visualization of the annotated data. The system is constantly extended to include additional annotation data and comparison methods.

**Conclusion:**

FACT serves as a highly flexible framework for the explorative analysis of large genomic and proteomic result sets. The program can be used online; open source code and supplementary information are available at .

## Background

A variety of algorithms and programs have been introduced to accomplish the processing of raw data as well as the statistical analysis of data from high-throughput experiments. But besides the mathematical complexity that needs to be handled, there is a biological complexity inherent to the data sets, too. Current means to analyze large-scale data sets usually target very specific questions and often fail to provide solutions that can be adapted to different types of data. Nevertheless, common and generalized questions for the interpretation of such data can be established as follows: i) What information is known about the analyzed features (clones, genes, e.g.)? ii) Are there correlations between the experimental outcomes and the additional information (shared pathways, etc.)? iii) Is the outcome comparable with results of other experiments (genomic or gene expression data sets, publications, etc.)?

The program *Flexible Annotation and Correlation Tool *(*FACT*) was developed to address these questions by integrating data sources, tools and algorithms in a single open framework. First, FACT allows merging information from various data sources into one comprehensive annotation for an experimental data set. It then provides functional analysis tools to inspect and correlate this heterogeneous information. The functionality of FACT can be extended through the inclusion of new data sources, algorithms and programs by defining additional modules from a prototype. This flexibility is achieved by a strong level of abstraction from the actual data, by the design of the underlying database and by the modular architecture of the software itself. The task to identify relevant biological interconnections reflected by the experimental results (e.g. participation of the analyzed genes in shared pathways) is what we are targeting with the software introduced here.

## Implementation

### Integration of data sources

The integration of bio-molecular data from diverse sources such as public databases or clinical parameters (*annotation*) is a key challenge in the process of the analysis of high-throughput experiments. While the interpretation of the outcome of a standard experiment used to depend on the knowledge of one human expert from the field, today's screening tools produce data quantities not manageable by human inspection. After receiving a list of differentially regulated genes from a microarray gene expression experiment comprising several hundreds or thousands of entries, it is not efficient to start the interpretation of these results by manually searching through publications. As a first step, broad biological themes should be identified and followed into a more detailed inspection.

Using network technologies, the availability of data sources is no more the limiting factor, but if accomplished manually, the obstacles for their integration are numerous. Often data are made available in different formats (HTML pages, flat files, direct database access) and very heterogeneous layouts. In addition the nomenclature (e.g. gene names) as well as the relationship of different systems to each other are often inconsistent and require many manual selection and modification steps. At the same time, as much knowledge as possible should be integrated about the data features analyzed, since interesting unknown pathways and interconnections might be hidden behind biological complexity.

**Figure 1:**
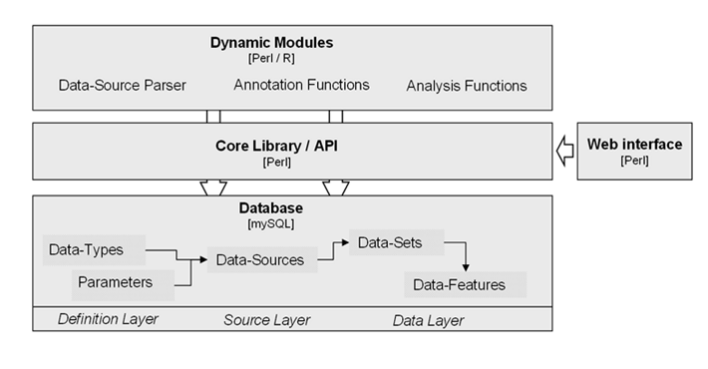
**Layered architecture of the FACT framework**. The database reflects the abstraction of any experimental or annotation data to *DataSets *with *DataFeatures*, originating from a specific *DataSource *for which *DataTypes *and *Parameters *have been defined. The core library (API) supplies all functionality for accessing the database and for the operation of diverse modules, which are adaptors for specific *DataSources *or functions. The web interface or other applications are using FACT API functions.

As the first aspect, FACT accomplishes the task of integrating heterogeneous information sources by abstraction from the specific data types to one basic concept. (figure 1, lower part). The smallest entities are *data features*, which are single items of information, either a name/value pair or additional textual description thereof. This could be a list of ids of clones with their relative expression as measured on a cDNA microarray. They are grouped into d*ata sets*, combining data features relating to the same experiment or a group of annotation terms for a certain set. In our example the clone/ratio pairs measured in one hybridization would be stored as one data set. The data sets originate from specific *data sources*, defining distinct types of experiments or annotation sources. One data source would be "cDNA microarray measurements with textual clone ids and numerical results". This architecture follows the idea, that the primary data must be represented at a sufficient level of abstraction to make the data independent of the source technology [1]. The concept applies to experimental as well as to annotation data. Meta-data about the different data sources is stored in dedicated tables of the underlying database, describing the source with date and type of data.

Differences in nomenclature and the problem of relating one type of experiment to an other, as the second obstacle for data integration, has been addressed for gene and protein centered research by the development of the GeneOntology system (GO) [2]. This hierarchical framework of a directed acyclic graph of annotations for gene attributes has become a *de facto *standard, which can be employed in the functional analysis of experiments. Similar projects have been initiated for example to organize the classification of molecular interactions in pathways and molecular complexes (Genome Knowledgebase / Reactome [3]). Using these resources FACT is able to compare experimental results from technically distant applications.

However, most experiments differ in focus and design and usually no standard solution for their interpretation can be applied. The third aspect for an integrating approach therefore is high flexibility concerning the application of diverse analysis methods that have already been developed for the interpretation of results or might be used in future.

### Architecture

**Figure 2:**
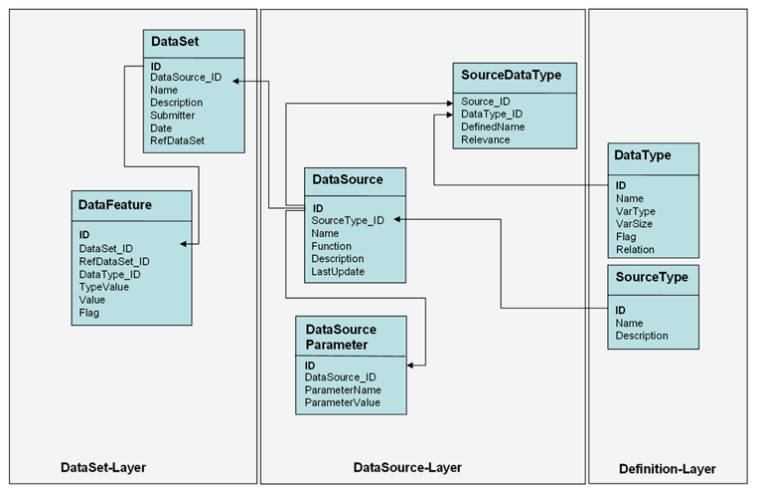
**FACT database schema**. The database schema reflects the generalized handling of heterogeneous data. At the definition layer the data sources are defined as experimental, annotational or analysis sources. Also the types of data that they use are specified here. These types are linked to the individual sources which are defined in the data source layer. Parameters that the functions handling the sources can take are stored as well. The actual data – experimental as well as annotational – are saved as data set and data features in the data set layer.

**Figure 3:**
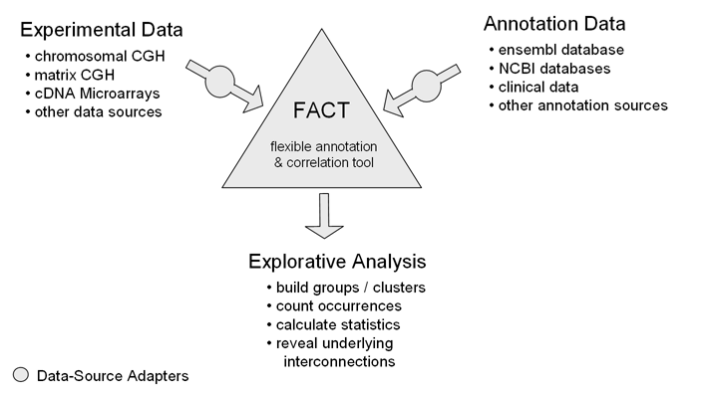
**Outline of the flow of information**. Modular DataSource-Adapters accomplish data access and data transformation from heterogeneous sources, making FACT a flexible framework.

FACT consists of a MySQL database, a core library (as an *Application Programming Interface*, API) and various modules written in the language Perl. We also created an interface for the web-based usage of all functions of FACT [4]. The database reflects the transformation of heterogeneous data into a generalized format, storing information in *DateSet *and *DataFeature *tables (figure 2). The core software framework supplies the basic functionality for data access in an object-oriented fashion and for adding and operating modules. These modules are *adaptors *and can be classified into three categories (figure 3):

#### I. Data loading

Different types of experimental data can be loaded using dedicated parser functions. This can be a simple functions to read tab-delimited data defined e.g. as a gene list with associated expression values. It can also be a more complex solution to handle case descriptions from comparative genomic hybridizations (CGH), a method that is employed to monitor copy numbers changes of all regions of a genome simultaneously on chromosome spreads. The individual modules read the specific file format, perform transformations to the generalized format (i.e. convert them into data features) and use core library functions to store the data.

#### II. Annotation

Varying data sources can be utilized for the annotation of experimental data sets by different data-access functions (e.g. GO terms for gene names). Modules achieve this for instance through access to an online database or to a local copy of such database. Data of interest are then gathered and stored.

#### III. Analysis

Different functions can be used to inspect the annotated information and highlight underlying patterns (e.g. overrepresented GO-terms). The modules typically produce textual and graphical output to draw the researcher's attention to the most promising features of his data.

Currently available functions of FACT are described below. Further flexibility is achieved by the concept of experimental and annotational data being reduced to the basic model of one *data set *with several *data features*, as described above.

All these modules use the FACT API. It offers a defined interface for the effortless extension to new sources and functions. Prototype modules for each category implementing this API are supplied. For the integration of annotation sources, available data can either be transferred to the local system (data warehousing) or linked to the original source (database federation); the FACT system allows both options to be used by the module functions. Currently remote databases are accessed by the *EnsEMBL*, *BBID *and *Reactome *modules; the *CpG *and *CGAP *functions use locally stored information (see below). The update of the local data is accomplished semi-automatically be invoking of the respective *update *function in the separate modules.

Finally, as there is an active development of software for the annotation and analysis of gene expression data in the language R (Bioconductor project [5]), and the handling of large data matrices is accomplished faster in R, we used the Perl/R interface *RSPerl *[6] in different modules to encapsulate analysis functions written in R. Other modules employ the functionality from the *BioPerl *[7] and *Ensembl *Perl API [8].

### Available functionality

A variety of modules for the handling of different data sources (table 1) as well as for the application of basic data analysis and display functions (table 2) were developed. Most of the current functionality is focused on handling human and murine gene annotation information. Additional functionality can be added in a "plug-and-play" fashion, since new modules can be loaded dynamically into FACT.

**Table 1:** Data Types and Sources accessible by current annotation modules.

**Data source, access method**	**Data provider, data location**	**Type of annotation used by FACT**
*Ensembl*, Perl API access to local or remote database	European Bioinformatics Institute and Wellcome Trust Sanger Institute (GB) [8],	Ensembl ID, Gene Symbol, Gene Name, Chromosomal Location, Homologues Genes, Interpro Domains, RefSeq Accession Number, Affymetrix ID
*euGenes*, local database	University of Indiana (USA) [10],	euGene ID, Gene Symbol, Gene Name, GDB ID, OMIM ID, Genomic Localization, GeneOntology Terms, Protein Accession Numbers
*Image Consortium*, local database	Lawrence Livermore National Laboratory (USA) [28],	Clone Image ID
*Biological Biochemical Image Database*, HTTP parser and HTTP request	National Institute of Aging, NIH (USA) [11],	Pathway Name and Image-link
*GeneOntology*, local database	GeneOntology Consortium [2],	ID and Name of GO-Term (Biological Process, Molecular Function, Cellular Localization)
*Cancer Genome Anatomy Project*, local database	National Cancer Institute, NIH (USA) [29],	Biocarta name, Biocarta short name, KEGG Pathway Name, KEGG Pathway ID, PFAM ID
*LocusLink */ *EntrezGene*, local database	NCBI/NIH (USA) [30], /	A. LocusLink ID, Gene Symbol, Gene Name, Genomic Localization, GeneOntology Terms, OMIM ID B. Key references (PubMed links)
*Mouse Genome Database*, local database	Jackson Laboratory (USA) [31],	MGI ID / Gene Symbol
Internal *CloneBase*, local database	Deutsches Krebsforschungs zentrum, Div. Molecular Genetics (D)	General Information on available Clones
*CpG*, local database	University of California Santa Cruz (USA),	Calculated relative CpG content of genomic region
*STRING*, local database	EMBL (D) [12], (medium or better confidence)	Protein interaction data (computed and imported from other databases)
*Affymetrix CEL *files	Affymetrix Inc. / FACT,	Use of Affymetrix probe IDs
*Reactome*, local database and HTTP request	European Bioinformatics Institute (GB) [3],	Pathway information

**Table 2:** Current data analysis and display modules

**Method Name**	**Reference**	**Method Description**
Simple Count	FACT	Count and display of occurrences of annotation terms
GO-Term Comparison	In part from *GO::TermFinder *[15]	Detection of significantly overrepresented GO terms in Gene List, based upon hypergeometric tail probability
MedLiner	*Bio::Biblio *(M. Senger, EBI)	List Publications with co-occurrences of terms
CGH database	Deutsches Krebsforschungs-zentrum, Div. Molecular Genetics (D)	Compare CGH results to archived data
goCluster	*goCluster*, G. Wrobel, available at	Detection of significantly overrepresented GO terms (based upon Fisher's exact test) in Clusters built with k-means algorithm
Hypergeometric Tail	In part from *GeneMerge *[14]	Detection of significantly overrepresented terms of any kind, based upon hypergeometric tail probability
CGH – Expression Comparison	FACT	Detect correlation between genomic and expression data sets, based on two-sided T-Tests
Chromosomal Plot	FACT	Display values or occurrences in genomic context

To read in experimental results, a simple list (with terms or term-value pairs) or table can be used, or more specialized parser functions can be employed to read and decipher the notations of different types of results. One parser (*2_Colums*) reads tab-, or semicolon-separated lists and stores the data as terms of the data type that is passed as a parameter (e.g. gene symbol or clone id) and the respective value. Another function (*LongList*) expects terms only (list of genes that are to be annotated) or Affymetrix probe ids (*AffyCelFile*). The parser for CGH results translates ISCN notations of cytogenetic alterations [9] into the distinct chromosomal bands that are affected while reading the data file. The bands are stored with the alteration -1 (loss of genomic material), +1 (gain), or +2 (high level amplification).

Data sources that can then be used for annotating these experimental results contain among others *EnsEMBL *databases [8], with functions providing numerous gene annotations on the human and murine genome (gene name, accession numbers, genomic localization, GO terms and other ids). The annotation data is fetched by using the EnsEMBL API or direct sql queries. Chromosomal locations expressed as cytogenetic bands can be translated in megabasepair positions. This can permit the direct comparison of results from genomic and expression experiments. Most common identifiers (IMAGE IDs, DDBJ/EMBL/GenBank accession numbers, international clone names, MGD (Mouse-Genome Database) IDs or Probe-IDs as used on expression microarrays in the Affymetrix system) are recognized and used by the different annotation modules. Additionally, homologous genes, sequence features, InterPro protein domains, CpG content and *PubMed *references can be acquired. The *euGenes *database [10] modules delivers an additional set of broad annotations (Gene Symbol and Name, GDB ID, OMIM ID, Genomic Localization, GO terms, etc.). The BBID module searches for representations of affected pathways in the *Biological Biochemical Image Database *[11]. We store links to the images which sometimes allow the clarification of interactions better than textual description alone. Additionally data is used from STRING [12] and Reactome [3] to point out protein interactions and involvement in molecular complexes.

Annotation steps can be concatenated, allowing deriving from specific (e.g. Affymetrix Probe-IDs) to more general terms (e.g. gene symbols). This broad annotation that is facilitated by FACT is crucial for the researcher to acquire a complete picture of his data. The user can export the combined lists of annotated data in HTML, XML or text format. If desired the system can send them by email.

**Figure 4:**
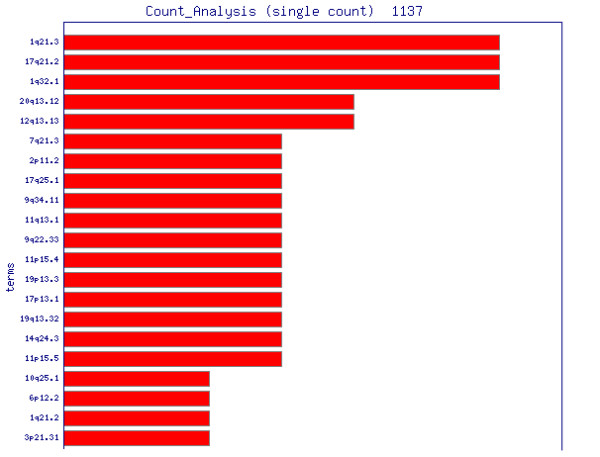
**Application of the FACT system for the functional analysis of microarray data of the development of non-melanoma skin cancer (*SimpleCount *function)**. Occurrences of annotation terms are counted and displayed to draw the researchers attention to potentially characteristic features of the data set. In this case the genomic bands at 1q21 seem to play an important role in the experiment.

**Figure 5:**
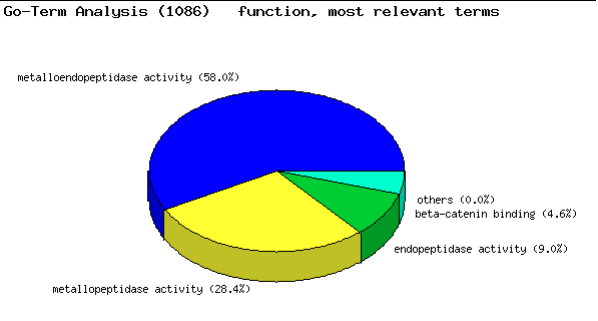
**Application of the FACT system in non-melanoma skin cancer research (*GoTerm *function)**. Overrepresented terms from a Gene Ontology annotation are displayed in a chart. The usage of the GO system is the most common approach for the functional interpretation of gene lists.

**Figure 6:**
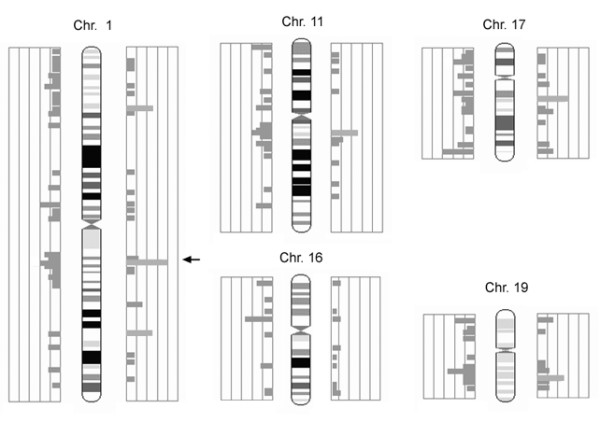
**Application of the FACT system in non-melanoma skin cancer research (*ChromPlot *function)**. Visual representation of the genomic distribution of analyzed features highlights the involvement of the genomic band 1q21 (first 5 chromosomes shown). In this case the localization of human homologues genes corresponding to murine clones over- and under expressed in squamous cell carcinoma are displayed.

Modules to correlate these annotated datasets with each other have been developed for FACT, incorporating existing algorithms or presenting new approaches (table 2). For example, a counting procedure (*SimpleCount*) reports the number of occurrences of each annotation term. The module is independent of the type of data, one or more data sets can be added up and a threshold for reporting can be defined. The results are displayed graphically in a chart and in a table format, directing the researcher's attention to distinct characteristics (figure 4). A more specific approach to interpret list of genes lies in the explicit usage of GO terms. As originally introduced by Khatri *et al*. in the program *OntoExpress *[13] there are different methods and implementation to search for those parts of the GO tree that appear more often in the gene list analyzed than by chance alone. For FACT we used an implementation from the *GeneMerge *program [14] and the GO::TermFinder perl module [15]. Occurrences of terms in a gene list are normalized against a background, which might contain the annotations of all terms spotted on a chip or the entire genome, by using the hypergeometric tail probability (with Bonferroni correction if desired, example: figure 5). In our implementation, the function can be applied on GO data as well as on any other kind of annotation to detect overrepresented terms. Alternatively the Fisher's exact test can be used for this purpose. We included part of this method taken from the *EASE *program [16] in a FACT function. We also developed a combined algorithm, which calculates a K-Means similarity matrix based on the experimental values and reports on significant terms with the Fisher's exact test within the identified groups afterwards (*goCluster*, G. Wrobel, available at ). Another analysis module dedicated to the comparison of genomic and expression data runs pairs of two-sided T-Tests on the groups of over- and under-expressed genes against the groups of enhanced/amplified and deleted genomic regions. This allows detecting a correlation between an altered genomic locus and the corresponding expression pattern. FACT can also represent experimental values or frequency counts in the genomic context which can be helpful for the identification and representation of localization effects in the genome. This is achieved by reading in the genomic locations of data sets and generating bar chart images on top of prepared chromosome ideograms (figure 6). The MedLiner module presents a simple literature mining tool which uses the Bio::Biblio perl functions to find citations that are shared by two or more gene (Figure 5). Example output files are also available at [4].

### Application of the program

The *Flexible Annotation and Correlation Tool *has proven especially helpful with the interpretation of results from genomic and expression microarray experiments, but most functions can be applied to a broad variety of experimental data.

We demonstrate the benefits of FACT for the functional interpretation of the results from a comprehensive analysis of gene expression patterns in the development of non-melanoma skin-cancer conducted within our group [L. Hummerich et al.: *Identification of novel tumor-associated genes in the process of squamous cell cancer development*; submitted]. Using two different sets of microarrays with 15.000 and 20.000 cDNA fragments respectively, the chemically induced multi-step development of squamous cell carcinoma was monitored. We used the dorsal skin of mice as a well-studied system for the development of epithelial cancer [18] with the carcinogen 7,12-dimethylbenz-[a]-anthracene and the tumor promoter 12-O-tetradecanoylphorbol-13-acetate as inducing agents. Expression values were measured at different time-points of tumor formation. Genes with differentiated expression patterns are expected to play a role in the development of human epidermal tumor development as well.

**Figure 7:**
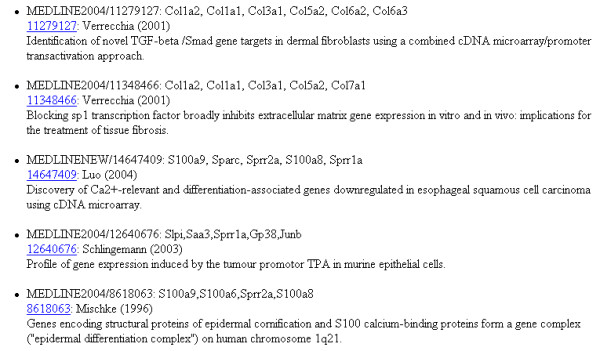
**Application of the FACT system in non-melanoma skin cancer research (*MedLiner *function)**. FACT's simple automated literature screen function displays publications mentioning groups of genes identified in the study (top 5 hits shown).

Preprocessed results where loaded into the FACT system as text files containing the murine clone identifier and expression values. Using mainly information from the *Ensembl *database, FACT annotation functions acquired corresponding gene names, genomic localizations, functional information from Gene Ontology, homologues human gene names and the genomic localization of those genes (supplement S1, complete set of annotated data). We applied different FACT analysis functions to explore the expression data (for example the *TermFinder *function to search for enriched functional groups of genes, figure 5). The function *SimpleCount *was used to search for enriched functional categories of annotations. Using the information of homologues genes, we were able to identify the human chromosomal band 1q21, as a region of accumulated differentially expresses genes in murine skin cancer formation (figure 4). The visual representation of the loci using the *ChromPlot *function highlights these findings (figure 6). With the *MedLiner *module we were to confirm that several regulated genes were collectively mentioned in previous publications (figure 7). A number of genes (S100A3, S100A6, S100A8, S100A9) which are part of the S100 family of calcium-binding proteins are involved in the regulation of AP-1 and NFκB-dependent transcription. Using FACT we were able to focus our analysis and to gain understanding of the relevance of the results from the microarray study. With the application, it was possible to find human homologues of the murine tumor-associated genes and to confirm the involvement of the S100 protein family in human epidermal malignancies.

### Comparison to and inclusion of other tools

There are several large-scale database projects that incorporate an immense spectrum of information about genes and gene product (*Ensembl*, *euGene*, *LocusLink/EntrezGene*, etc.). The *Ensembl *project for example also allows the user to display his own selected data sources in the context of the full genome annotation through the *Distributed Annotation System *[19], and it offers the possibility of mining the data of several genomes using the *EnsMart *software [20]. FACT uses *Ensembl*, *LocusLink/EntrezGene *and *euGene *data and complements them with other annotation resources; it allows the user to apply different analysis functions on the combined data.

Recently, a variety of computational tools have been introduced to aid in the interpretation of results, some of which are of interest concerning FACT. The majority of the programs use GO annotations to gain an interpretation of gene expression data. *OntoExpress *[13] was introduced in 2002 and offers the options to use hypergeometric, chi-square, binomial and Fischer's exact test to score annotation term derived from gene lists. It also allows the appliance of different methods (False Discovery Rate (FDR), Bonferroni, Holm, Sidak) for the multiple experiment correction [17]. The program can also include KEGG pathway information and chromosomal localization and is now part of the Onto-Tools collection to offer further functionality [21]. *EASE *[16]*/ DAVID *[22] offer a broad variety of annotation options in their latest version including all major database identifiers, protein domain and pathway information. The Fisher's exact test is used for the detection of enriched terms. *GoMiner *[23] and numerous other tools listed at the GO website [24] can be used for the annotation of gene lists with GO terms. *GeneMerge *[14] uses the hypergeometric tail probability with Bonferroni correction to test GO terms, genomic localizations and KEGG pathway information and is used in parts within the FACT system. FACT uses the available Perl code of *GO::TermFinder *[15] for the GO annotation and the detection of significantly overrepresented terms using the FDR. It also includes a function combining K-means clustering and Fisher's exact test. *GEPAS *[25] and *GECKO *[26] are two recently introduced large software packages that include functional analysis and visualization steps. In contrast to FACT they are focused on the initial statistical evaluation and on the analysis of gene expression microarray data. GFINDer [27] is a system that offers annotations on GO, pathway information, protein domains and genetic disorders. It analyses with count functions and appropriate tests (Hypergeometric, Binomial, Fisher's, Z or Poisson Test).

This list of available tools is far from complete and not all aspects are covered. The FACT system was developed with the focus to include and extend the functionality of tools like these. To our knowledge, the individual programs do not offer the same degree of flexibility and openness to different data sources and analysis methods. New functions can be added to FACT by simply uploading the respective module. The system is designed as an open framework for the explorative analysis using a variety of methods on annotational data. It is not restricted to or focused on Gene Ontology-based interpretation or the analysis of gene expression data alone and should facilitate the development and application of new analysis approaches. The system is constantly being extended to include additional aspects. With the submission as an open-source project we want to encourage other researchers to participate in this development.

## Conclusion

To gain a more complete picture of results obtained from high-throughput experiments such as DNA-microarrays, automated procedures are required for annotation and analysis. At the same time it is usually a matter of testing and not known beforehand, which of the possible approaches for the functional analysis will be the most informative or appropriate. The *Flexible Annotation and Correlation Tool *offers the flexibility to integrate and compare annotation data and different algorithms in one environment by using a unified data basis. Data sets of different nature and format can be incorporated, diverse analytical algorithms can be applied and the user can add his own data integration and analysis functions. As a flexible framework for the explorative meta-analysis of genomic, proteomic or other experiments, FACT can help with the task of analyzing the biological complexity, allowing researchers to bridge gaps between different kinds of experiments and acquiring a more complete interpretation of large-scale experiments.

## Availability and requirements

- **Project name: **Flexible Annotation and Correlation Tool (FACT).

- **Project home page: **

- **Operating system: **tested on Linux SUSE 9.1

- **Programming language: **Perl (5.8.1)

- **Other requirements: **MySQL database (4.0.15); for specific modules: R (1.8.0 with RSPerl) and Bioconductor (1.4.0); for full installation: Apache web-server (apache2-prefork-2.0.48); additional Perl modules: BioPerl (1.2.1), Ensembl (currently 28). Please refer to website for full listing.

- **License: **Open Source GNU GPL (see licence document)

- **Any restrictions to use by non-academics: **written licence needed

## Abbreviations

API – application programming interface, CGH – comparative genomic hybridization, FACT – Flexible annotation and correlation tool, GO – Gene Ontology, ISCN – international system for human cytogenetic nomenclature

## Authors' contributions

FK designed and implemented the FACT system and the web-interface, ND re-designed and extended it, GW helped with the initial design and supplied R-modules, LH carried out the microarray experiments and supplied additional ideas, GT conducted the integration into other analysis systems, PL supervised the FACT project. All authors read and approved the final manuscript.
